# Evaluating the Bayesian causal inference model of intentional binding through computational modeling

**DOI:** 10.1038/s41598-024-53071-7

**Published:** 2024-02-05

**Authors:** Takumi Tanaka

**Affiliations:** Graduate School of Humanities and Sociology and Faculty of Letters, The University of Tokyo, Tokyo, Japan

**Keywords:** Human behaviour, Computational models, Computational neuroscience, Cognitive neuroscience, Sensorimotor processing, Sensory processing

## Abstract

Intentional binding refers to the subjective compression of the time interval between an action and its consequence. While intentional binding has been widely used as a proxy for the sense of agency, its underlying mechanism has been largely veiled. Bayesian causal inference (BCI) has gained attention as a potential explanation, but currently lacks sufficient empirical support. Thus, this study implemented various computational models to describe the possible mechanisms of intentional binding, fitted them to individual observed data, and quantitatively evaluated their performance. The BCI models successfully isolated the parameters that potentially contributed to intentional binding (i.e., causal belief and temporal prediction) and generally better explained an observer’s time estimation than traditional models such as maximum likelihood estimation. The estimated parameter values suggested that the time compression resulted from an expectation that the actions would immediately cause sensory outcomes. Furthermore, I investigated the algorithm that realized this BCI and found probability-matching to be a plausible candidate; people might heuristically reconstruct event timing depending on causal uncertainty rather than optimally integrating causal and temporal posteriors. The evidence demonstrated the utility of computational modeling to investigate how humans infer the causal and temporal structures of events and individual differences in that process.

## Introduction

Time and causality are intertwined in the brain. Temporal relationships between events tell us about the causal structures in the world^1,2^, whereas causal belief often distorts our subjective time^3–7^. A well-known causality-driven illusion is intentional binding—the compression of the time interval between an action and its consequence. In a typical method^8^, this effect is assessed by comparing two conditions. In the operant condition, one’s action (e.g., keypress) causes the presentation of a stimulus (e.g., tone). In the baseline condition, the same events occur by themselves in separate trials. Consequently, the observers perceive the action to be performed later and the stimulus to be presented earlier in the operant condition than in the baseline one. Named *intentional* binding, this effect is originally thought to be specific to (or at least prominent in) intentional action and its sensory outcome^9^. When the movement was triggered by an external magnetic stimulation to the brain, Haggard et al.^8^ observed the repulsion, not compression, of the perceived interval. Moreover, compression was no longer observed when the observer was led to believe that there was no causality between the two events^10^. Intentional binding therefore has been utilized as a proxy for the sense of agency^11^; however, its interpretation has sparked considerable debate. Recent studies have questioned the necessity of intentionality in this effect. Similar temporal compression has been observed for outcomes of externally induced^12–14^ or forced^15,16^ actions and for unintended outcomes^17^. Moreover, the essentiality of movement in intentional binding has been challenged by findings showing temporal attraction between externally triggered causal events^5,18,19^, between the inhibition of an action and its consequences^20^, and even between the actions of others and their outcomes^21^. These discoveries have prompted some scholars to propose that temporal compression may be indicative of a broader mechanism of causality perception, thus favoring the term “temporal binding” or “causal binding”^5,18^. The connection between intentional binding and the sense of agency is still disputed, with some studies confirming their correlation^22^, while others do not^23^. This ongoing debate highlights the need for elucidation of the fundamental nature and underlying mechanisms of intentional binding^24,25^.

Multisensory integration framework has attracted growing attention as a potential explanation for intentional binding^26,27^. Humans are consistently inundated with noisy signals from the external world. To optimize perception in such a situation, the Bayesian brain integrates multiple signals weighted by their reliability. Computationally, this integration serves to minimize the variance of final estimates, known as Maximum-Likelihood Estimation (MLE)^28^. In this context, intentional binding occurs when one estimates the timing of an event (e.g., action) by considering the timing of another event (e.g., tone). Supporting this notion, amplifying the sensory noise around motor or outcome events, thereby reducing their reliability, resulted in the timing estimates of these events being more heavily biased towards the timing of the other, more reliable event^26,29–31^. However, MLE explains only how, but not when, signals are integrated. Importantly, the perceptual system cannot unconditionally merge available signals because integrating irrelevant signals worsens estimation. Addressing this problem, the recent Bayesian Causal Inference (BCI) model elucidates the cognitive processes by which the brain infers the causal structure—determining which signals originate from the same source—through the synthesis of prior knowledge and sensory evidence^32–34^. When one detects a remarkable (temporal or spatial) discrepancy between events, it breaks down the perceived causality and thereby integration^35^.

Bαsed on the BCI model, Legaspi and Toyoizumi^36^ implemented a computational model of intentional binding. In the multisensory contexts, integration is generally driven by the *unity prior* that different signals should represent an identical source in time and space^37,38^. This assumption may not be appropriate in the case of intentional binding where the action and outcome are usually separated by a detectable (typically 250 ms) gap^29^. Therefore, Legaspi and Toyoizumi^36^ generalized the model by introducing a free parameter, ${\mu }_{AO}$, that represents the expectation for the interval length between the *Action* and *Outcome*. An ideal observer represents the probability of experiencing a certain action-outcome interval, which biases temporal estimation. Such a flexible prior for temporal correlations is called *the coupling prior*^37,38^, as opposed to the unity prior for simultaneity. Importantly, their model predicts either repulsion or compression depending on whether the interval between sensory signals is shorter or longer than the prior. Thus, their model can explain the repulsion in an involuntary situation as well as compression^8^. I refer to this model as the Legaspi and Toyoizumi (LT) model in the current study.

Computational modeling has multiple benefits^39^. It outputs probabilistic predictions of an observer’s responses (e.g., time estimates) given sensory inputs. Comparing these predictions with observed data allows one to quantitatively evaluate the model’s ability. One can also compare the performance of different models and gain insight into the internal processes that produce the responses. Furthermore, estimating the model parameters specifies (the source of) interindividual and intergroup variation. While intentional binding is generally a robust phenomenon, large individual differences are linked to specific psychiatric disorders such as schizophrenia^40–42^. Examining internal parameters may provide cues on what characterizes one’s binding tendency.

Despite these potential advantages, the LT model’s validity has not been sufficiently examined thus far. Legaspi and Toyoizumi^36^ reproduced tendencies reported in two significant studies^8,31^ but did not fit their model to observations at either individual or trial-by-trial levels. Moreover, although Legaspi and Toyoizumi^36^ validated their model by referring to the involuntary condition in^8^, it is unclear what process the external stimulation modulated, e.g., attention, motor execution, or causal cognition. It is thus worth testing the LT model in a typical voluntary condition. Finally, they considered only one model (i.e., the LT model) and did not compare it with other types of BCI models with different algorithms or those with non-Bayesian alternatives.

Therefore, the current study quantitatively evaluated the computational models of intentional binding based on empirical data. I measured intentional binding using a classic clock method. Instead of employing an atypical involuntary condition, I manipulated signal intervals by varying the physical size of action-outcome gaps. As mentioned above, the LT model that is based on a coupling prior predicts repulsion rather than compression when the signal interval is sufficiently short. Technically, this manipulation was required to isolate the causal and temporal priors, which both influence the binding effects^36^. Implementing various computational models, I first examined their ability to distinguish their free parameters via parameter recovery. Then, for the first time, I fitted the models to observed data. Based on the model fit and estimated parameter values, I discuss the possible mechanisms underlying intentional binding.

## Results

### Behavioral results

I conducted an online experiment (N = 76) based on a typical intentional binding task (Libet clock paradigm; see “Method” for details). The experiment was developed based on the JavaScript plugin with which Galang et al.^43^ replicated robust intentional binding. In separate blocks, observers performed arbitrary keypress and/or heard a tone while viewing a clock rotating on a screen, and they estimated the timing of either of the events based on the clock-hand position. In the baseline tasks, they estimated the timing of the keypress or tone presentation that occurred independently. The operant tasks included three conditions of the action-outcome interval; observers’ keypress randomly caused a tone after 0 ms, 250 ms, or 500 ms. Estimation errors were calculated by subtracting the actual time of a keypress or tone from the reported time of the corresponding event.

**Figure 1:**
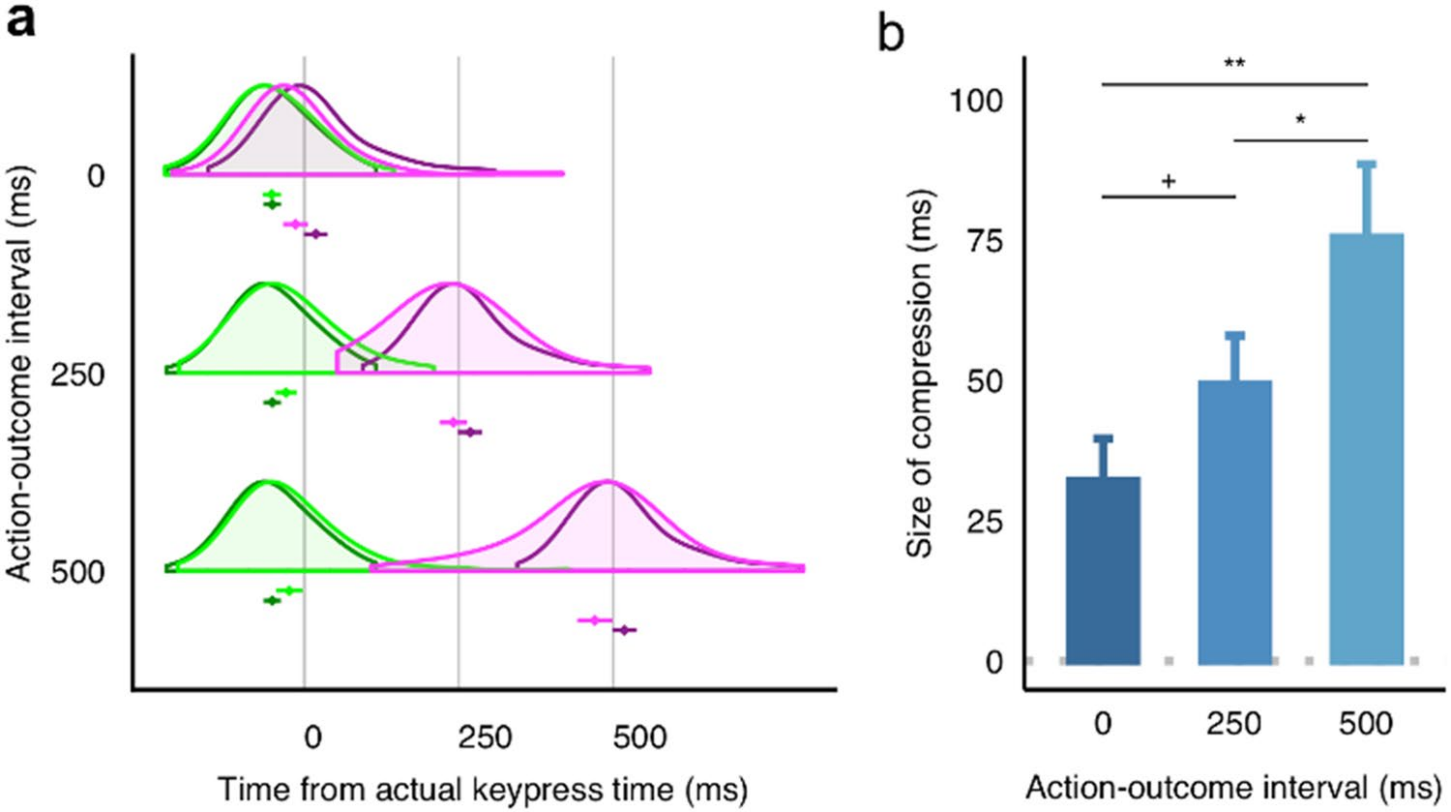
Behavioral results of the experiment. (**a**) The distributions and means of the time estimates (the dots with error bars below the distribution plots) were reported in each action-outcome interval condition (0, 250, or 500 ms). The x-axis represents the timeline, with the actual time of keypress marked as zero. The time estimates are represented by color-coded plots: green for keypress estimates and purple for tone estimates. Lighter shades of these colors represent estimates in the operant condition, while darker shades denote baseline estimates. For ease of comparison, the single baseline estimates for keypress and tone are replicated across each interval condition. This is done by adjusting their offsets, allowing for a direct visual comparison with the operant condition estimates in each interval. (**b**) The mean magnitude of the time compression (i.e., intentional binding) as a function of action-outcome interval length. The sizes of the compressions were calculated by summing up the perceptual shifts from the baseline for the keypress and for the tone (see Methods for details). The symbols above the pairs of bars indicate significance in the post-hoc pairwise *t*-tests: **p < 0.01, *p < 0.05, and ^+^*p* < 0.10. All error bars represent standard errors for within-participants design^44^.

The distributions of the estimates are illustrated in Fig. 1a. The baseline estimates for the keypress and tone timing were respectively biased in anticipatory and lagged ways (keypress baseline: mean = -52.22, SD = 61.56; tone baseline: mean = 18.27 ms, SD = 61.56 ms). The estimation errors from the analysis of variance (ANOVA) revealed the main effects of the conditions (baseline, 0 ms operant, 250 ms operant, or 500 ms operant) for both keypress ($F(\mathrm{3,225})=16.04$, $MSE=1029.85$, $p<0.001$, ${\widehat{\eta }}_{G}^{2}=0.028$) and tone timing ($F(\mathrm{3,225})=10.66$, $MSE=\mathrm{2,855.29}$, $p<.001$, ${\widehat{\eta }}_{G}^{2}=0.030$). Compared to the baseline condition, the keypress timing was overestimated in the operant trials with the 250 ms (*t*(75) = 4.59, *p* < 0.001, *d* = 0.30) and 500 ms (*t*(75) = 3.94, *p* = 0.001, *d* = 0.31) action-outcome intervals but not in the 0 ms (*t*(75) = − 0.21, *p* = 0.833, *d* = 0.01) interval. There were also significant differences between 0 and 250 ms (*t*(75) = 6.63, *p* < 0.001) and 0 ms and 500 ms (*t*(75) = 4.23, *p* < 0.001), but not between 250 and 500 ms (*t*(75) = 1.30, *p* = 0.394). In contrast, the tone timing was underestimated compared to the baseline in all the interval conditions (0 ms: *t*(75) = − 4.54, *p* < 0.001, *d* = 0.38; 250 ms: *t*(75) = -3.60, *p* = 0.002 *d* = 0.29; and 500 ms: *t*(75) = -4.76, *p* < 0.001, *d* = 0.42). While there was no significant difference in the timing estimations between the 0 ms and 250 ms conditions (*t*(75) = 0.74, *p* = 0.464), and between the 0 ms and 500 ms conditions (*t*(75) = − 1.25, *p* = 0.427), a significant difference was observed between the 250 and 500 ms conditions (*t*(75) = − 3.65, *p* = 0.002). In summary, I confirmed the biases in time estimation consistent with typical intentional binding (i.e., compression) in all the conditions except for the action in the 0 ms condition.

Summing up action and outcome binding, I plotted the overall intentional binding in Fig. 1b. Perceived intervals were compressed in proportion to the lengths of the physical intervals ($F(\mathrm{2,150})=7.53$, $MSE=\mathrm{4,793.56}$, $p=0.001$, ${\widehat{\eta }}_{G}^{2}=0.040$). Although the difference between the 0 and 250 ms conditions was not statistically significant (*t*(75) = 1.77, *p* = 0.080, *d* = 0.24), the 500 ms condition elicited greater compression than the 0 ms (*t*(75) = 2.87, *p* = 0.011, *d* = 0.46) and 250 ms (*t*(75) = 3.43, *p* = 0.003, *d* = 0.23) conditions. One-sample t-tests confirmed the occurrence of compression in all the intervals (0 ms: $t(75)=4.27$, $p<0.001$, *d* = 0.48; 250 ms: $t(75)=5.60$, $p<0.001$, *d* = 0.64; and 500 ms: $t(75)=5.75$, $p<0.001$, *d* = 0.65).

### Computational modeling

#### Introduction of models

To explore the mechanism that produced these behavioral results, a computational modeling analysis was performed. Here, I explored twelve computational models as potential explanations for intentional binding. As the details for the model algorithms, fitting, and comparison are described in the “Methods” section, I briefly introduce the concepts of each model.

Eight of the twelve models were variations of the BCI accounts in which an observer infers the causal and temporal structures of events based on their prior expectations and sensory evidence. These models assume that observers have two priors regarding causal and temporal relationships between the action and the tone, respectively. The causal prior, $P\left(\xi =1\right),$ represents the prior probability of a causal relationship between an action (i.e., keypress) and subsequent events (i.e., tone). The temporal prior depends on the causal belief. The observers have an expectation for the interval between causally linked keypress and tone, which plays a role as joint prior of keypress and tone timing. Here, this prior is assumed to be a Gaussian distribution with mean ${\mu }_{AO}$ and standard division (SD) ${\sigma }_{AO}$. For an observer with a unity prior, ${\mu }_{AO}$ approaches zero. Meanwhile, for the acausal events that occur independently, there should be no relationship between event timing and therefore the prior for event interval would be flat (i.e., a uniform distribution) instead of a Gaussian distribution. The likelihood is calculated as the probability at which the occurrence of an event is signaled to the brain at a given time. For causal and acausal cases, one can shape the posterior distribution for event timing by combining the temporal prior and the likelihood function and obtain the candidates of time estimation as the peak location of the distribution (maximum-a-posteriori; MAP). Therefore, temporal estimation does not fully depend on when sensory signals arrive but is subject to their expected intervals for the likely causal scenario.

To draw final estimates, an observer can employ several strategies on how to utilize the two potential estimates depending on causality. The LT model^36^ directly compares the posterior probabilities of estimates (i.e., MAP) between causal and acausal cases and identifies more likely estimates. This corresponds to adopting the MAP estimates of the joint posterior of causal scenario and event timing. In contrast, multisensory research has proposed that causal inference and timing estimation can be hierarchically performed; the brain first infers causality by calculating the marginal posterior probability and then explores the likely event timing accordingly^45^. We consider three strategies respectively called *model-selection*, *model-averaging*, and *probability-matching*^46^. Model-selection refers to a strategy where the observer selects the most likely causal scenario (either the presence or absence of a causal relationship) based on the posterior probabilities and then makes estimations in this chosen scenario. This is different from model-averaging, where the observer takes into account both causal and acausal scenarios, averaging the estimates across these scenarios weighted by their respective posterior probabilities. Probability-matching, on the other hand, is another strategy where the observer randomly chooses between the causal and acausal scenarios based on their posterior probabilities for each trial, thereby *matching* the probability distribution of the scenarios. In summary, the BCI variants comprised two components, i.e., two types of temporal prior (i.e., the unity and coupling priors) and four estimation strategies. Factorially combining them resulted in the eight BCI models.

In contrast to the BCI models, I expressed a mandatory integration model without causal inference by fixing $P(\xi =1)$ to one. In this model, the signals are always integrated regardless of their timing, which means simple MLE. Because the four estimation strategies above make no difference when $P(\xi =1)=1$, there were only two types of mandatory integration models with the coupling and unity priors, respectively.

The remaining two models represent non-Bayesian accounts. First, I assessed a fixed-criterion model in which the observer provided temporal judgments by averaging the timing of the action and outcome whose temporal gap was smaller than a certain threshold $\phi$^47^. Alternatively, the false-report model assumes that observers can falsely report the non-target event instead of the target event with a certain probability, ${P}_{fr}$. For instance, when an observer estimates the outcome timing in some trials of the action operant condition, the mean action estimate apparently shifts toward the outcome, and vice versa. Although averaging the data including these erroneous responses can produce apparent binding-like results, such a risk has been overlooked in previous studies.

These 12 models above predict estimates for keypress and tone timing in operant tasks based on the distribution of the estimates in the baseline tasks. In the baseline tasks, as there is only one event present, the observers simply make time estimates based on the sensory inputs. Therefore, one can regard the distribution of time estimation in baseline tasks as the distribution of sensory noises. The mean and variance of this baseline distribution are included in all 12 models as the fixed parameters. Moreover, the models can be evaluated relative to a baseline null model in which observers derive estimates similarly in the operant and baseline tasks and thus no binding occurs.

#### Parameter recovery

**Figure 2:**
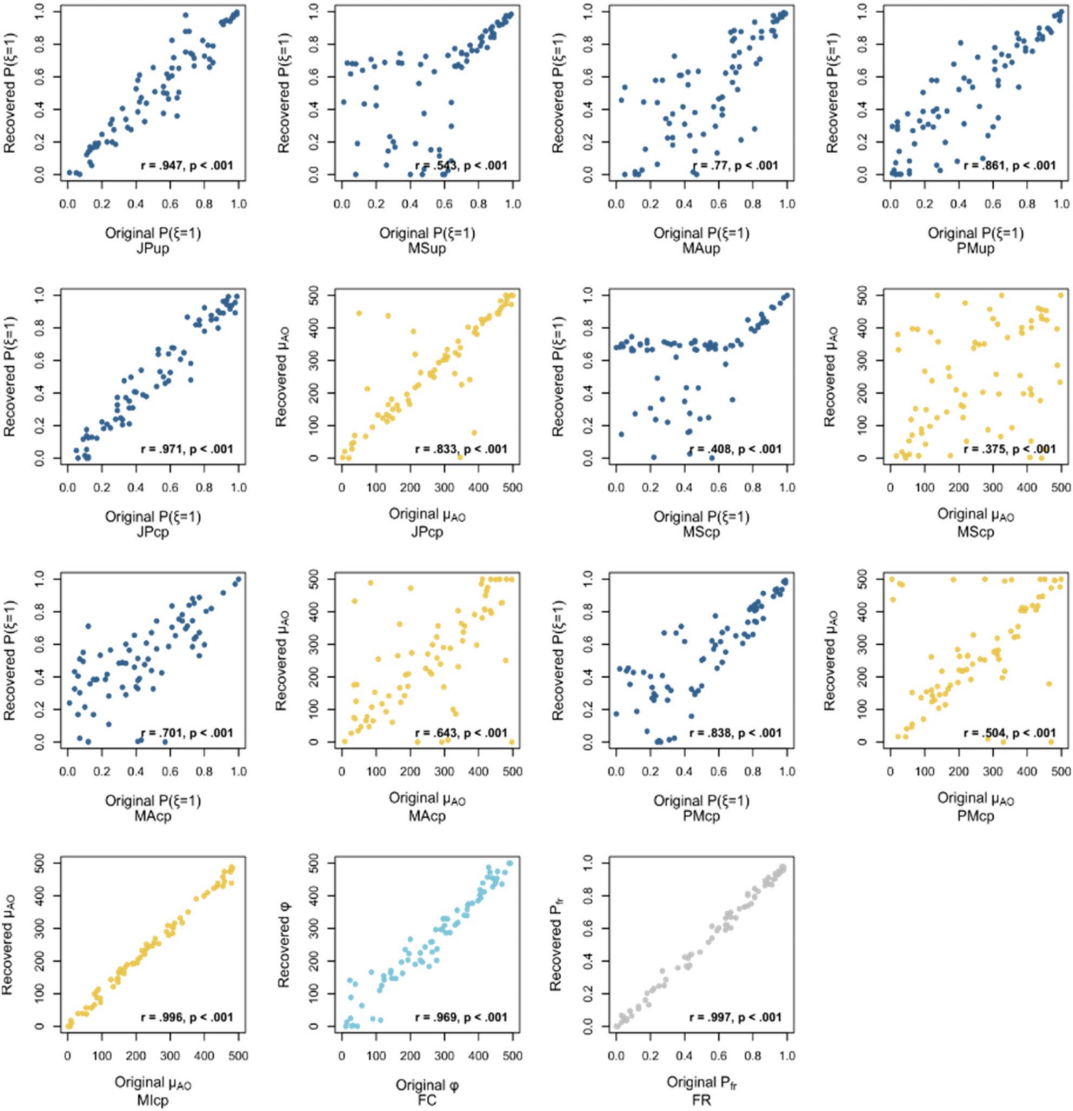
Results of parameter recovery. The relationships between original (randomly sampled) and recovered parameter values. Pearson’s correlation coefficients, *r*, and *p*-values in the tests for no correlation are shown for each pair. The words under each plot indicate model names. The capital characters in the model names indicate the algorithms (JP: BCI with joint posterior; MS: BCI with model-selection; MA: BCI with model-averaging; PM: BCI with probability-matching; MI: mandatory integration; FC: fixed-criterion model; and FR: false-report model). For the BCI models, the lowercase characters indicate the type of temporal prior: up: unity prior; and cp: coupling prior.

First, I investigated whether the models could identify their parameters via parameter recovery. Each simulated model was fitted to the data for 76 ideal observers whose free parameters were randomly sampled from the possible ranges (see “Methods” for details). The original and recovered parameter values were generally robustly correlated (mean *r* = 0.74, *ts* > 3.48, *ps* < 0.001, see Fig. 2 for details). Except for the BCI models with model-selection, all the models recovered their parameters with high accuracy. The model-selection strategy always rejected the integration when the causal posterior was lower than a certain threshold, which seemed to make it difficult to isolate the parameters. For more detailed results, including all the values of original and recovered parameters, see Supplementary Table S1.

#### Model recovery

**Figure 3:**
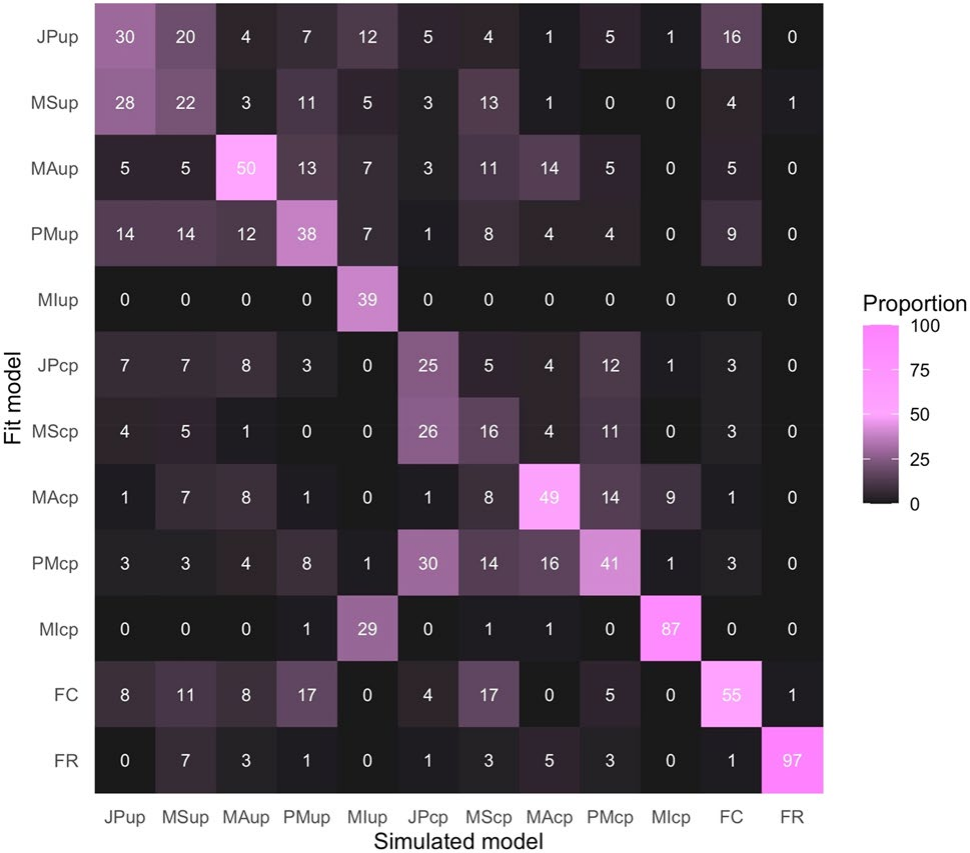
Model Recovery Matrix. This heatmap visualizes the cross-comparison of fit for 12 distinct models applied to datasets simulated by the same suite of models. Each cell indicates the percentage of datasets for which the row model provided the best explanation of the column-simulated data, with the color intensity reflecting the proportion (from 0 to 100, as denoted by the color scale on the right). Shades closer to pink signify a higher percentage, emphasizing the instances of accurate model recovery. The diagonal dominance suggests that models tend to best fit the data they generated, highlighting the discriminative power and fidelity of the modeling approach. See the Fig. 2 caption for the meaning of the model names’ acronyms.

I also conducted a comprehensive model recovery analysis to assess the discriminative ability of the 12 computational models. The analysis involved fitting each model to datasets generated by these same models obtained in the earlier parameter recovery. The fitting was evaluated based on the Akaike information criterion (AIC), which considers both the model's goodness of fit and its complexity. Figure 3 presents a model recovery matrix, which visualizes the comparative effectiveness of each model in predicting the datasets generated by itself and by the other models. The heatmap revealed a general trend where models accurately identified data from their own simulations, as indicated by the prominent diagonal line. The analysis generally demonstrated good discrimination between Bayesian models and non-Bayesian models such as mandatory integration, fixed-criterion, and false-report. However, the matrix also revealed instances of less successful model discrimination. Especially, the Bayesian models with the joint posterior strategy and those with the model-selection strategy showed a less distinct separation, indicating a challenge in differentiating between the data they produced. The observed overlaps in discrimination underscore the need for careful interpretation in subsequent analyses.

#### Fitting results

The models were fitted to each observer’s data at the individual level and to the pooled data at the group level. In each case, I estimated the best free parameters using MLE.

**Figure 4:**
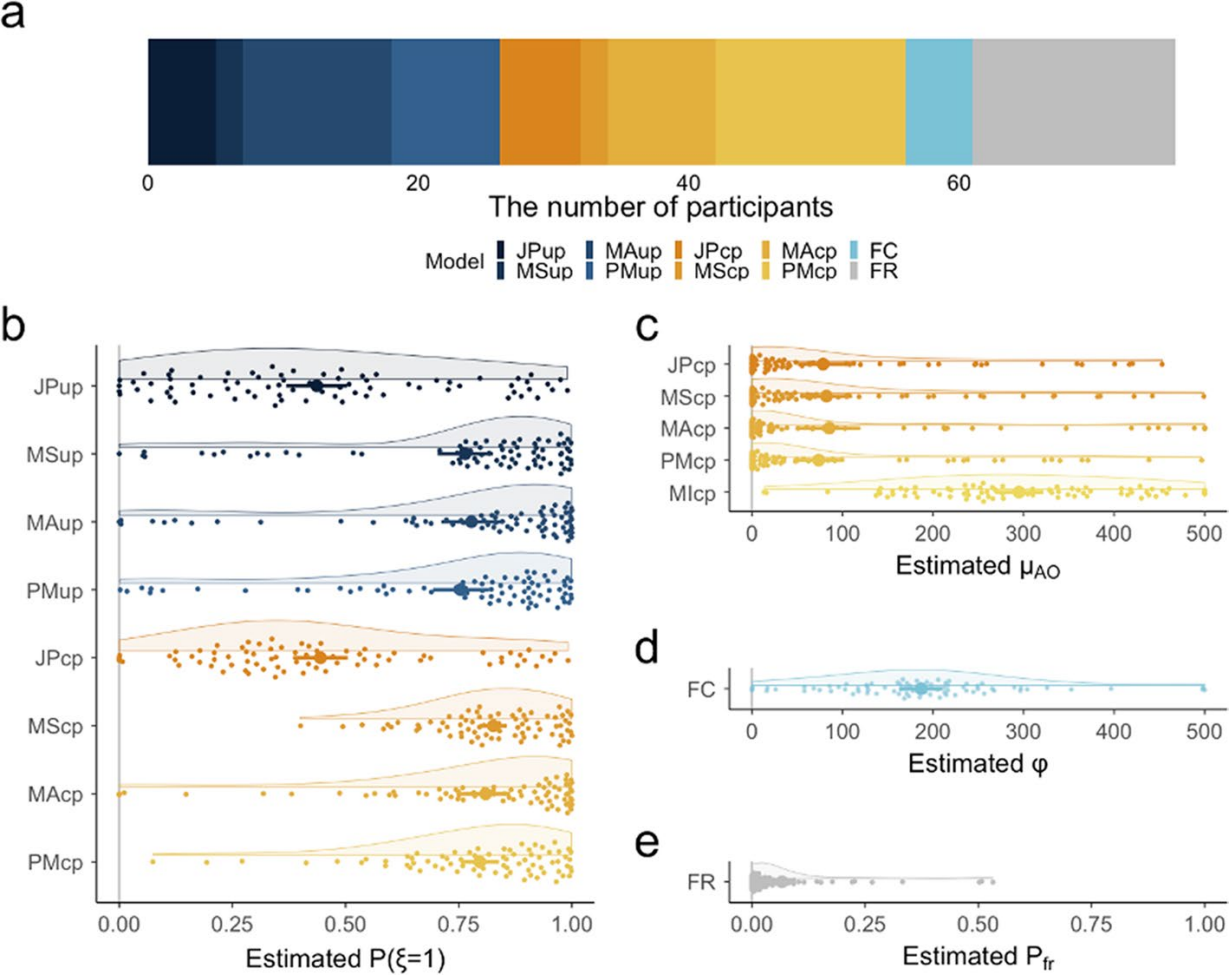
Results of individual-level model fitting. (**a**) The portfolio of models that best accounted for individual observers’ data (76 observers in total). (**b**) Distribution of estimated values of $P(\xi =1)$ for each type of BCI model. Small dots correspond to individual observers and large dots correspond to mean values. Error bars represent standard errors for within-participants design^44^. (**c**) Distribution of the estimated values of ${\mu }_{AO}$ for models with the coupling prior. (**d**) Distribution of the estimated values of $\phi$ in the fixed-criterion model. (**e**) Distribution of the estimated values of ${P}_{fr}$ in the false-report model. See the Fig. 2 caption for the meaning of the model names’ acronyms.

The best models for individual observers are shown in Fig. 4a. The AIC indicated that the BCI models best explained 56 out of 76 observers’ data, whereas the fixed-criterion model yielded the best fits for only five observers (for all the AIC values for each model for each observer, see Supplementary Table S2). No observer was best explained by the mandatory integration models. At the individual level, there was no dominant BCI strategy shared by the observers. Of the 56 observers, 26 preferred the BCI models with the unity prior, and 30 preferred those with the coupling prior. Lumping different priors, 11 observers preferred the BCI models with the joint posterior, 22 the probability-matching, 19 the model-averaging, and 4 the model-selection. I plotted the model parameters’ estimated values in Fig. 4b–e. The estimated $P(\xi =1)$ tended to be smaller in the BCI models based on the joint posterior than in other BCI algorithms (Fig. 4b). While Legaspi and Toyoizumi^36^ assumed $P(\xi =1)$ of 0.9 in the voluntary condition, the mean of the estimated value in the LT model (i.e., the BCI model based on the coupling prior and joint posterior) was ~ 0.5. Moreover, the mean (± SD) of ${\mu }_{AO}$ was 95.66 (± 94.32) ms in the LT model and 117.44 (± 130.09) ms in the BCI models overall (Fig. 4c), which were also smaller than previously suggested^36^. The mean (± SD) of estimated $\phi$ was 186.65 (± 104.03) in the fixed-criterion model (Fig. 4d). Surprisingly, the remaining 15 observers’ estimations were best accounted for by the false-report model (Fig. 4a). Probably due to these observers, the group-level analysis for all pooled data identified the false-report model as the best (see Supplementary Table S3 for detailed results). The model’s best fitting parameter was ${P}_{fr}=0.04$, implying that observers made erroneous estimates in ~ 4% of all the trials, on average (see also Fig. 4e).

Considering that the 15 observers whose estimates were best accounted for by the false-report model might not appropriately perform the task, as planned (see pre-registration), I conducted another group-level fitting to the data excluding them. The results of this fitting are shown in Table 1, including the estimated parameter values, log-likelihood (LL), and AIC for each candidate model. They reveal that the probability-matching BCI model with the unity prior and that with the coupling prior almost equally better account for the remaining data. According to Hilbe^48^, an AIC difference of less than 2.5 can be interpreted as nonsignificant. With an AIC difference of ~ 10 from these two models, the BCI based on the joint posterior and fixed-criterion model yield the next-best fits. Notably, only the mandatory integration models showed lower performance than the null model.

**Table 1:** Results of group-level fitting after excluding the observers whose responses followed the false-report model.

Model	P (ξ = 1)	μ_AO_	φ	P_fr_	Log-likelihood	AIC
JPup	0.204	0	-	-	− 131,281.18	262,566.36
MSup	0.786	0	-	-	− 131,293.32	262,590.63
MAup	0.728	0	-	-	− 131,359.65	262,723.31
PMup	0.693	0	-	-	− 131,276.07	262,556.15
MIup	1	0	-	-	− 260,633.01	521,268.03
JPcp	0.202	0	-	-	− 131,296.44	262,598.88
MScp	0.769	0.119	-	-	− 131,297.52	262,601.04
MAcp	0.759	0	-	-	− 131,355.05	262,716.10
PMcp	0.702	0.046	-	-	− 131,275.16	262,556.31
MIcp	1	271.642	-	-	− 171,466.64	342,937.29
FC	-	-	198.837	-	− 131,297.61	262,599.22
FR	-	-	-	0.018	− 131,299.45	262,602.89
Null	-	-	-	-	− 132,020.34	264,042.67

Values in parentheses represent the fixed parameters. Hyphens indicate the parameters that are not included in the model. See the Fig. 2 caption for the meaning of the model names’ acronyms.

## Discussion

BCI has been considered a promising account for intentional binding. While the theory has recently been formulated as a computational model, it has not been thoroughly tested. In this study, I implemented a range of computational models, including both the BCI and non-BCI models, and quantitatively evaluated their ability. Instead of employing an involuntary condition, I manipulated the physical action-outcome interval length to test the model prediction. Although some partial problems remain, which will be discussed below, parameter recovery and model recovery ensured the validity of the model at a certain level. This allowed me to fit the models to observed data for the first time.

### The mechanism underlying intentional binding

The parameter estimation and model comparison provided several implications for the underlying mechanism for intentional binding. Most of the observers were well explained by variants of BCI models, whereas some were explained by potential irregular responses. Notably, the BCI models outperformed the simple MLE as well as the fixed-criterion models at both individual and group levels. This indicates that humans do not always integrate an action and subsequent event but decide whether and how to integrate them by inferring their causal relationship.

I considered various BCI algorithms. While there was no consensus among individuals, probability-matching showed the best performance at the group level. In probability-matching, one sequentially infers the causal scenario and event timing, which is different from inference based on a joint posterior in the LT model^36^. Interestingly, probability-matching is a computationally suboptimal strategy compared to model-averaging^46^. As the calculation of the joint posterior and model-averaging are expensive, humans may heuristically reconstruct event timing depending on causal uncertainty. This is likely, given that probability-matching is known to be predominantly used in several perceptual and cognitive tasks^49–53^. It should be noted, however, that some Bayesian model algorithms, such as the probability-matching model with the coupling prior and the LT model, did not discriminate each other very well (see Fig. 3). Refinement of the models and experiments with larger sample sizes and more repetitions may contribute to drawing more robust conclusions.

In addition to the causal prior, the BCI models have the temporal prior regarding the action-outcome interval. The estimated mean values of temporal prior (${\mu }_{AO}$) were distributed around zero, with some variance across individual observers (Fig. 4c). The group-level fitting also estimated it at close to zero (see Table 1); therefore, introducing the coupling prior did not clearly improve model performance compared to using the unity prior. Indeed, although the coupling-prior model predicts illusory repulsion when signals inform a shorter interval than the prior, the behavioral results confirmed general binding effects (i.e., compression) even in the 0 ms condition. These data suggest that, contrary to the assumption in the LT model, people tend to believe that their actions will immediately cause a consequence. Intentional binding, at least for an average observer, can be explained by unity integration as in classical multisensory models^37,38^.

Nevertheless, setting the priors as free parameters is advantageous as it allows for a flexible description of binding tendencies. The current study confirmed substantial individual differences in the estimated parameters. Moreover, the causal and temporal priors depend on the task contexts. For example, the parameter for the causal prior should change by manipulating the action-outcome contingency^54–56^. Additionally, people can learn the temporal structure of a task^57–60^. Matute et al.^61^ also randomly presented 0 ms or 500 ms action-outcome intervals and revealed that the time estimate of the action shifted away from the outcome as more trials were experienced. One can reason that this was because the experience of delays led to larger time intervals being predicted. Consistent with this idea, when the interval length was blocked (not randomized), Glazebrook et al.^62^ observed strong binding for the 0 ms interval. It is thus possible that, in many studies that fix the interval to 250 ms, the prediction may approach that value (e.g., 230 ms), as Legaspi and Toyoizumi suggested^36^. However, importantly, the current analysis estimated the temporal prior at approximately zero, despite the presence of 250 and 500 ms conditions, indicating the robustness of the unity assumption as a prior^63^.

According to the BCI models, the fact that intentional binding reflects both causal belief and temporal prediction may lead to difficulty in interpretation. Computational modeling can isolate these factors and explain binding tendencies in a certain individual and situation. It may help researchers investigate which (cognitive or sensory) processes are responsible for aberrant binding, for example, in schizophrenia^40–42^. This approach may also be useful to explore the relationship between cognitive and sensory processes. While the current models assume dependency of temporal prior on causal inference, causal prior may also be subject to the temporal context. Although such influences could not be independently quantified in the current setting, future research can investigate their dynamics by manipulating causal and temporal contexts. Furthermore, contrary to the traditional analysis of binding, the computational models can provide trial-by-trial (probable) predictions of whether one would causally bind action and outcome. By assessing if this prediction would coincide with explicit causal judgments, researchers can explore a novel, powerful tool to investigate the sense of agency.

### Possibility of non-Bayesian accounts

In principle, my findings support the idea that the BCI account can explain intentional binding. However, it is important to note that a substantial number of observers were better explained by a misunderstanding of the task demand, implying a potential problem with the typical measurements of intentional binding. This does not negate the occurrence of intentional binding in these observers but suggests that incidental errors might have inflated the observed binding effect. The classic clock task is usually designed to minimize such a risk by asking for action and outcome time reports in separate sessions. When the target events were mixed within a block, the binding effect was indeed enhanced^64^. A critical limitation of traditional mean value analysis is its inability to differentiate between erroneous estimations and actual perceptual binding. As shown in this study, the computational modeling approach can help researchers manage such a risk.

### Limitations

As a theoretical limitation of the present study, there may be other models that are better than those considered here. This study did not cover all possible accounts for intentional binding. Although I focused on models that assumed a common mechanism for perceptual shifts of keypress (action binding) and tone (outcome binding), some researchers have questioned this idea. For example, Tonn et al.^65^ observed no correlation between the magnitude of action binding and that of outcome binding across individuals (see also^25^). I also did not find similar changes in action and outcome binding as a function of interval length. This evidence raises the question of the validity of the overall compression as an index and suggests independent mechanisms for two subcomponents. Hon^66^ provided a theoretical explanation for action and outcome binding, attributing them to attentional processes and predictive processes respectively. Nevertheless, to the best of my knowledge, there is currently no theory that independently provides quantitative predictions for each type of binding. Future studies are expected to build a computational model that explains the dissociation of the two binding types and compare it with the common mechanism model.

Furthermore, there are several practical limitations. First, the clock paradigm was used to measure intentional binding; however, other tasks, such as direct interval estimation^18,19,21,22,67–70^, can also be used to measure this effect. Given that these tasks possibly engage different processes^25,71^, the current findings may not be applicable to other measurements. Additionally, this study was conducted in an online setting, which may have introduced uncontrolled noise such as latency in tone presentation and variability in tone timing. These factors may have increased the length or uncertainty of the event interval. I believe the risk of these noises was minimized by manipulating the action-outcome intervals for an observer and incorporating individual baseline differences into the model. Nonetheless, it is worth noting that the absolute values of the estimated parameter (e.g., ${\mu }_{AO}$) should be interpreted with caution. Moreover, the present study only quantified the mean, but not the variance, of the temporal prior distribution. It was difficult to estimate the variance while differentiating it from other parameters, such as $P(\xi =1)$, in the current design^36^. It is possible that there are also individual differences in the variance of this distribution, with some observers having a strong expectation for a specific interval length while others do not have such an expectation. Given that the perceived order of actions and outcomes is unlikely to be reversed, future models might consider asymmetric distributions like log-normal or gamma distributions for the temporal prior instead of the Gaussian distribution.

## Conclusion

In conclusion, this study provides the first demonstration of fitting the computational models for intentional binding to trial-by-trial observation. The findings support the idea that people use BCI to recognize causal and temporal event structures. Additionally, probability-matching emerged as a possible candidate for implementing BCI. These results provide insight into the mechanisms underlying intentional binding and highlight the usefulness of computational modeling in understanding this phenomenon. Adopting this computational approach may also enable future research to characterize action-outcome binding patterns in both healthy and clinical populations by quantifying their causal and sensory priors.

## Methods

### Computational model

All the models shared the assumption regarding how ideal observers generate estimates in the baseline condition but differed in the assumptions in the operant condition. Here, I define the actual timing of an action as the onset time (${t}_{A}^{*}=0$). Although the tone occurs by itself in the baseline condition, I express the actual time of an outcome, ${t}_{O}^{*}$, by the corresponding action-outcome interval, ${t}_{AO}$ (e.g., 250 ms), to compare with that in the operant condition (${t}_{O}^{*}={t}_{A}^{*}+{t}_{AO}$). The letters A and O in the parameter names represent 'action' and 'outcome,' respectively, while the asterisk denotes the actual value (physical timing). Our time perception is not precise in itself and is subject to internal noise and mechanical bias. The initial percept is thus expected to follow a Gaussian distribution whose mean is biased from the actual time and whose variance is the level of internal noises, namely,

1 $$\begin{array}{c}\begin{array}{cc}& {\tau }_{A}\sim N\left({t}_{A}^{*}+{b}_{A},{\sigma }_{A}^{2}\right)\\ & {\tau }_{O}\sim N\left({t}_{O}^{*}+{b}_{O},{\sigma }_{O}^{2}\right)\end{array}\end{array}$$

where ${b}_{A}$ and ${b}_{O}$ represent biases, ${\sigma }_{A}$ and ${\sigma }_{O}$ represent noises, and ${\tau }_{A}$ and ${\tau }_{O}$ represent event timing for action and outcome, respectively.

The task requires an *inverse inference* to find the most likely timing of action ${\widehat{t}}_{baselineA}$ and outcome ${\widehat{t}}_{baselineO}$ from the corresponding noisy signals, ${\tau }_{A}$ and ${\tau }_{O}$, respectively. A Bayesian observer solves this by exploring the timing of action ${t}_{A}$ and outcome ${t}_{O}$ that maximize the likelihoods given by the conditional probabilities,

2 $$\begin{array}{c}\begin{array}{cc}& P\left({\tau }_{A}|{t}_{A}\right)=\frac{1}{\sqrt{2\pi }{\sigma }_{A}}\mathit{exp}\left(-\frac{({\tau }_{A}-{t}_{A}{)}^{2}}{2{\sigma }_{A}^{2}}\right)\\ & P\left({\tau }_{O}|{t}_{O}\right)=\frac{1}{\sqrt{2\pi }{\sigma }_{O}}\mathit{exp}\left(-\frac{({\tau }_{O}-{t}_{O}{)}^{2}}{2{\sigma }_{O}^{2}}\right)\end{array}\end{array}$$

As Eq. (2) takes on its maximum values when ${\tau }_{A}={t}_{A}$ and ${\tau }_{O}={t}_{O}$,

3 $$\begin{array}{c}\begin{array}{c}{\widehat{t}}_{baselineA}={\tau }_{A}\\ {\widehat{t}}_{baselineO}={\tau }_{O}\end{array}\end{array}$$

Therefore, one can regard Eq. (1) as the distributions of baseline estimates and can obtain the approximation of the parameters, ${b}_{A}$, ${\sigma }_{A}^{2}$, ${b}_{O}$, and ${\sigma }_{O}^{2}$, from observation (see “Results”).

### Models based on Bayesian causal inference

In the operant condition, the observers experience two events, an action and an outcome. A Bayesian observer integrates these different signals by weighting their reliability to obtain the best estimates. Details can be found in Ref.^36^; however, in summary, their BCI model further assumes that such integration occurs only when the observers believe the two events were correlated, that is, the action caused the outcome. The observer is thus challenged by two intertwined problems: when events occurred and whether they were causal. An optimal strategy maximizes the posterior probability, $P({t}_{A},{t}_{O},\xi |{\tau }_{A},{\tau }_{O})$, that depends on the causal case, $\xi$. The binary parameter, $\xi$, indicates whether the action and outcome are relevant ($\xi =1$) or not ($\xi =0$). From the Bayesian theorem,

4 $$\begin{array}{c}P\left({t}_{A},{t}_{O},\xi |{\tau }_{A},{\tau }_{O}\right)=\frac{P\left({\tau }_{A},{\tau }_{O}|{t}_{A},{t}_{O},\xi \right)P\left({t}_{A},{t}_{O},\xi \right)}{P\left({\tau }_{A},{\tau }_{O}\right)}.\end{array}$$

Given that the initial percepts, ${\tau }_{A}$ and ${\tau }_{O}$, are independent of the causality $\xi$, $P\left({\tau }_{A},{\tau }_{O}|{t}_{A},{t}_{O},\xi \right)=P\left({\tau }_{A}|{t}_{A}\right)P\left({\tau }_{O}|{t}_{O}\right)$. Given $P({t}_{A},{t}_{O},\xi )$ is a joint distribution of ${t}_{A}$, ${t}_{O}$, and $\xi$, $P\left({\tau }_{A},{\tau }_{O}|{t}_{A},{t}_{O},\xi \right)P\left({t}_{A},{t}_{O},\xi \right)=P\left({\tau }_{A}|{t}_{A}\right)P\left({\tau }_{O}|{t}_{O}\right)P\left({t}_{A},{t}_{O}|\xi \right)P\left(\xi \right).$ Thus, the numerator that determines the peak location (i.e., maximum likelihood estimate) can be decomposed as follows:

5 $$\begin{array}{c}P\left({\tau }_{A},{\tau }_{O}|{t}_{A},{t}_{O},\xi \right)P\left({t}_{A},{t}_{O},\xi \right)=P\left({\tau }_{A}|{t}_{A}\right)P\left({\tau }_{O}|{t}_{O}\right)P\left({t}_{A},{t}_{O}|\xi \right)P\left(\xi \right).\end{array}$$

The conditional probabilities, $P({\tau }_{A}|{t}_{A})$ and $P({\tau }_{O}|{t}_{O})$, are likelihoods of sensory signals given event timing and are given by Eq. (1). The $P({t}_{A},{t}_{O}|\xi )$, the prior of event timing, depends on the causality. When the action and outcome are correlated ($\xi =1$), their gap should be normally distributed and, otherwise ($\xi =0$), the prior distribution should be the uniform distribution, that is,

6 $$\begin{array}{c}P\left({t}_{{\text{A}}},{t}_{{\text{O}}}\mid \xi \right)=\left\{\begin{array}{ll}\frac{{\text{exp}}\left(-\frac{{\left({t}_{{\text{O}}}-{t}_{{\text{A}}}-{\mu }_{{\text{AO}}}\right)}^{2}}{2{\sigma }_{{\text{AO}}}^{2}}\right)}{\sqrt{2\pi }{\sigma }_{AO}T}& \left(\xi =1\right)\\ \frac{1}{{T}^{2}}& \left(\xi =0\right)\end{array}\right.\end{array}$$

where ${\mu }_{AO}$ is a prior for the action-outcome interval and ${\sigma }_{AO}$ is its standard deviation. Moreover, $T$ represents the finite integral ranges for ${t}_{A}$ and ${t}_{O}$ to normalize the probability distribution. Following^36^, the fixed parameters, ${\sigma }_{AO}$ and $T$, were set to 10 ms and 250 ms, respectively. Note that ${\mu }_{AO}$ is a free parameter in the model with the coupling prior, whereas it is fixed to zero in the model with the unity prior.

Combining Eqs. (2) and (6), an observer obtains the optimal estimates that maximize the posterior probability (i.e., the MAP estimates) in each causal scenario, Eq. (5). In the causal case,

7 $$\begin{array}{c}\begin{array}{c}{\widehat{t}}_{A,\xi =1}={\tau }_{{\text{A}}}+\frac{{\sigma }_{{\text{A}}}^{2}}{{\sigma }_{{\text{tot}}}^{2}}\left({\tau }_{{\text{O}}}-{\tau }_{{\text{A}}}-{\mu }_{{\text{AO}}}\right)\\ {\widehat{t}}_{O,\xi =1}={\tau }_{{\text{O}}}-\frac{{\sigma }_{{\text{O}}}^{2}}{{\sigma }_{{\text{tot}}}^{2}}\left({\tau }_{{\text{O}}}-{\tau }_{{\text{A}}}-{\mu }_{{\text{AO}}}\right)\end{array}\end{array}$$

with the total variance, ${\sigma }_{tot}^{2}={\sigma }_{A}^{2}+{\sigma }_{O}^{2}+{\sigma }_{AO}^{2}$. In the acausal case, like the baseline estimates, the peak locations are computed simply based on $P({\tau }_{A}|{t}_{A})$ and $P\left({\tau }_{O}|{t}_{O}\right):$

8 $$\begin{array}{c}\begin{array}{c}{\widehat{t}}_{A,\xi =0}={\tau }_{A}\\ {\widehat{t}}_{O,\xi =0}={\tau }_{O}\end{array}\end{array}$$

There are several possible ways to determine the final estimates using these conditional estimates. In the model proposed by Legaspi and Toyoizumi^36^, one prefers the MAP of the joint posterior of causality and event timing. Because the estimates, ${t}_{{\text{A}}}$ and ${t}_{{\text{O}}}$, at the peaks are known as Eqs. (7) and (8), the ratio of the peak values can be computed:

9 $$\begin{array}{c}r\equiv \frac{\underset{{t}_{{\text{A}}},{t}_{{\text{O}}}}{{\text{max}}}P\left({t}_{{\text{A}}},{t}_{{\text{O}}},\xi =1\mid {\tau }_{{\text{A}}},{\tau }_{{\text{O}}}\right)}{\underset{{t}_{{\text{A}}},{t}_{{\text{O}}}}{{\text{max}}}P\left({t}_{{\text{A}}},{t}_{{\text{O}}},\xi =0\mid {\tau }_{{\text{A}}},{\tau }_{{\text{O}}}\right)}.\end{array}$$

The observer finally takes the estimates for the causal case with the higher peak (MAP);

10 $$\begin{array}{c}\begin{array}{cc}{\widehat{t}}_{operantA}& =\left\{\begin{array}{l}{\widehat{t}}_{A,\xi =1}\mathrm{ if }r>1\\ {\widehat{t}}_{A,\xi =0}\mathrm{ if }r\le 1\end{array}\right.\\ {\widehat{t}}_{operantO}& =\left\{\begin{array}{l}{\widehat{t}}_{O,\xi =1}\mathrm{ if }r>1\\ {\widehat{t}}_{O,\xi =0}\mathrm{ if }r\le 1\end{array}\right.\end{array}\end{array}$$

In addition, I implemented hierarchical strategies for causal inference. Instead of the MAP of the joint posterior, $P({t}_{A},{t}_{O},\xi |{\tau }_{A},{\tau }_{O})$, the observer may use the marginal probability of each causal scenario, given by

11 $$\begin{aligned} & P\left( {\xi = 1|\tau _{{\text{A}}} ,\tau _{{\text{O}}} } \right) = \frac{{P\left( {\tau _{{\text{A}}} ,\tau _{{\text{O}}} ,\xi = 1} \right)}}{{P\left( {\tau _{{\text{A}}} ,\tau _{{\text{O}}} ,\xi = 1} \right) + P\left( {\tau _{{\text{A}}} ,\tau _{{\text{O}}} ,\xi = 0} \right)}} \\ & P\left( {\xi = 0|\tau _{{\text{A}}} ,\tau _{{\text{O}}} } \right) = 1 - P\left( {\xi = 1|\tau _{{\text{A}}} ,\tau _{{\text{O}}} } \right) \\ \end{aligned}$$

with

12 $$\begin{aligned} P\left( {\tau _{{\text{A}}} ,\tau _{{\text{O}}} ,\xi = 1} \right) & = \int {\int\limits_{R} {P\left( {\xi = 1|t_{{\text{A}}} ,t_{{\text{O}}} ,\tau _{{\text{A}}} ,\tau _{{\text{O}}} } \right)dt_{{\text{A}}} dt_{{\text{O}}} } } \\ & = \frac{{P\left( {\xi = 1} \right)\sigma _{{{\text{AO}}}} }}{{\sqrt {2\pi } \sigma _{{AO}} T\sigma _{{{\text{tot}}}} }}{\text{exp}}\left( { - \frac{{\left( {\tau _{{\text{O}}} - \tau _{{\text{A}}} - \mu _{{{\text{AO}}}} } \right)^{2} }}{{2\sigma _{{{\text{tot}}}}^{2} }}} \right) \\ \end{aligned}$$

and

13 $$\begin{aligned} P\left( {\tau _{{\text{A}}} ,\tau _{{\text{O}}} ,\xi = 0} \right) & = \int {\int\limits_{R} {P\left( {\xi = 0|t_{{\text{A}}} ,t_{{\text{O}}} ,\tau _{{\text{A}}} ,\tau _{{\text{O}}} } \right)dt_{{\text{A}}} dt_{{\text{O}}} } } \\ & = \frac{{P\left( {\xi = 0} \right)}}{{T^{2} }}. \\ \end{aligned}$$

here, three strategies were considered to produce the final estimate. With the model-selection strategy, the observers consider only the most likely case:

14 $$\begin{aligned} & \hat{t}_{{operantA}} = \left\{ {\begin{array}{*{20}l} {\hat{t}_{{A,\xi = 1}} \;{\text{if}}\;P\left( {C = 1\left| {\tau _{A} ,\tau _{V} } \right.} \right) > 0.5} \hfill \\ {\hat{t}_{{A,\xi = 0}} \;{\text{if}}\;P\left( {C = 1\left| {\tau _{A} ,\tau _{O} } \right.} \right) \le 0.5} \hfill \\ \end{array} } \right. \\ & \hat{t}_{{operantO}} = \left\{ {\begin{array}{*{20}l} {\hat{t}_{{O,\xi = 1}} \;{\text{if}}\;P\left( {C = 1\left| {\tau _{A} ,\tau _{O} } \right.} \right) > 0.5} \hfill \\ {\hat{t}_{{O,\xi = 0}} \;{\text{if}}\;P\left( {C = 1\left| {\tau _{A} ,\tau _{O} } \right.} \right) \le 0.5} \hfill \\ \end{array} } \right. \\ \end{aligned}$$

Meanwhile, with the probability-matching strategy, the use of estimates is stochastically determined in accordance with the probability of each case:

15 $$\begin{aligned} & \hat{t}_{{operantA}} = \left\{ {\begin{array}{*{20}l} {\hat{t}_{{A,\xi = 1}} \;{\text{if}}\;P\left( {\xi = 1\left| {\tau _{A} ,\tau _{O} } \right.} \right) > p} \hfill \\ {{\text{where}}\;p \in \left[ {0:1} \right]\;{\text{uniform}}\;{\text{distribution}}} \hfill \\ {\hat{t}_{{A,\xi = 0}} \;{\text{if}}\;P\left( {\xi = 1\left| {\tau _{A} ,\tau _{O} } \right.} \right) \le p} \hfill \\ {{\text{and}}\;{\text{sampled}}\;{\text{on}}\;{\text{each}}\;{\text{trial}}} \hfill \\ \end{array} } \right. \\ & \hat{t}_{{operantO}} = \left\{ {\begin{array}{*{20}l} {\hat{t}_{{O,\xi = 1}} \;{\text{if}}\;P\left( {\xi = 1\left| {\tau _{A} ,\tau _{O} } \right.} \right) > p} \hfill \\ {{\text{where}}\;p \in \left[ {0:1} \right]\;{\text{uniform}}\;{\text{distribution}}} \hfill \\ {\hat{t}_{{O,\xi = 0}} \;{\text{if}}\;P\left( {\xi = 1\left| {\tau _{A} ,\tau _{O} } \right.} \right) \le p} \hfill \\ {{\text{and}}\;{\text{sampled}}\;{\text{on}}\;{\text{each}}\;{\text{trial}}} \hfill \\ \end{array} } \right. \\ \end{aligned}$$

Finally, while the two strategies above exclusively employ either the causal or acausal case in a trial, the model-averaging strategy integrates estimates based on causal and acausal cases by weighting their likelihood:

16 $$\begin{aligned} & \hat{t}_{{operantA}} = P\left( {\xi = 1\left| {\tau _{A} ,\tau _{O} } \right.} \right)\hat{t}_{{A,\xi = 1}} + P\left( {\xi = 0\left| {\tau _{A} ,\tau _{O} } \right.} \right)\hat{t}_{{A,\xi = 0}} \\ & \hat{t}_{{operantO}} = P\left( {\xi = 1\left| {\tau _{A} ,\tau _{O} } \right.} \right)\hat{t}_{{O,\xi = 1}} + P\left( {\xi = 0\left| {\tau _{A} ,\tau _{O} } \right.} \right)\hat{t}_{{O,\xi = 0}} \\ \end{aligned}$$

### Fixed-criterion model

As an alternative to the BCI model, the fixed-criterion model proposes that the observer simply averages the timings of action and outcome signals when their interval is below a certain threshold, $\phi :$

17 $$\begin{aligned} & \hat{t}_{{operantA}} = \left\{ {\begin{array}{*{20}l} {\frac{{\tau _{A} + \tau _{O} }}{2}\;{\text{if}}\;\left( {\tau _{O} - \tau _{A} } \right) \le \phi } \hfill \\ {\tau _{A} \;{\text{if}}\;\left( {\tau _{O} - \tau _{A} } \right) > \phi } \hfill \\ \end{array} } \right. \\ & \hat{t}_{{operantO}} = \left\{ {\begin{array}{*{20}l} {\frac{{\tau _{A} + \tau _{O} }}{2}\;{\text{if}}\;\left( {\tau _{O} - \tau _{A} } \right) \le \phi } \hfill \\ {\tau _{O} \;{\text{if}}\;\left( {\tau _{O} - \tau _{A} } \right) > \phi } \hfill \\ \end{array} } \right. \\ \end{aligned}$$

### False-report model

In the false-report model, the observer in the operant condition accidentally reports the timing of another event rather than the to-be-reported one at a certain probability, ${P}_{fr}$:

18 $$\begin{aligned} & \hat{t}_{{operantA}} = \left\{ {\begin{array}{*{20}l} {\tau _{A} \;{\text{if}}\;P_{{fr}} > p} \hfill \\ {{\text{where}}\;p \in \left[ {0:1} \right]\;{\text{uniform}}\;{\text{distribution}}} \hfill \\ {\tau _{O} \;{\text{if}}\;P_{{fr}} \le p} \hfill \\ {{\text{and}}\;{\text{sampled}}\;{\text{on}}\;{\text{each}}\;{\text{trial}}} \hfill \\ \end{array} } \right. \\ & \hat{t}_{{operantO}} = \left\{ {\begin{array}{*{20}l} {\tau _{O} \;{\text{if}}\;P_{{fr}} > p} \hfill \\ {{\text{where}}\;p \in \left[ {0:1} \right]\;{\text{uniform}}\;{\text{distribution}}} \hfill \\ {\tau _{A} \;{\text{if}}\;P_{{fr}} \le p} \hfill \\ {{\text{and}}\;{\text{sampled}}\;{\text{on}}\;{\text{each}}\;{\text{trial}}} \hfill \\ \end{array} } \right. \\ \end{aligned}$$

### Null model

The baseline model assumes that the observers similarly estimate the timing in the baseline and operant conditions:

19 $$\begin{array}{c}\begin{array}{cc}& {\widehat{t}}_{operantA}={\widehat{t}}_{baselineA}={\tau }_{A}\\ & {\widehat{t}}_{operantO}={\widehat{t}}_{baselineO}={\tau }_{O},\end{array}\end{array}$$

predicting no intentional binding.

## Experiment

### Ethical considerations

This study was approved by the institutional review board of the University of Tokyo (no. 202119) and was conducted in accordance with the ethical standards of the 1964 Declaration of Helsinki. All observers provided informed consent prior to the commencement of the experiments.

### Deviations from pre-registration

The experimental methods, including the sample size, procedures, and exclusion criteria were pre-registered (https://aspredicted.org/rc8y6.pdf). Although this included the analysis plan for summarized behavioral data, I changed the ANOVA design because I noticed that the registered one (2*3 repeated-measure ANOVA) was not consistent with the experimental design. The computational modeling analysis was exploratory. Only the rough plan was pre-registered in which I would compare models using the AIC by fitting the data at the group and individual levels and excluding participants whose responses would best be explained by either the false-report model or the null model.

### Observers

I set 80 observers as the target sample size. According to Galang et al.^43^, this sample size is sufficient to detect action and outcome shifts with typical effect sizes reported in a meta-analysis^25^. Assuming that data from some observers would be excluded from the analysis due to incomplete commitment and systematic issues, I invited 100 observers via an online crowdsourcing service (CrowdWorks; https://crowdworks.jp/). Eighty-three participants appropriately completed the experiment on time and I analyzed the data from 76 observers, excluding five left-handed ones and two with low performance (33 females; mean ± SD of age = 41.28 ± 9.01).

### Procedure

The experimental procedure was almost the same as that in the web-based experiment by Galang et al.^43^, except that there were three levels of the action-outcome interval. The experiment was conducted on a web browser with a JavaScript application and a jsPsych plugin^72^. Observers viewed a clock rotating on a screen and estimated the timing of the target event based on the position of the clock hand. There were four types of tasks across 2 (measurement: baseline or operant) measurements by 2 (target event: keypress or tone) events. In the action baseline task, observers pressed the space bar and reported the timing of the keypress. In the outcome baseline task, they heard a tone externally generated at a random point between 1280 and 3840 ms from the trial onset and estimated its timing. In the operant action/outcome task, observers pressed a key and heard the tone followed by a certain interval, and they estimated the key/tone timing. The action-outcome interval was pseudo-randomly chosen from 0 ms, 250 ms, or 500 ms. Note that, considering the possibility that a longer duration of outcome tone introduces temporal variability, an auditory tone with a duration of 50 ms was used, instead of the 200 ms tone employed by Galang et al.^43^. Observers performed different tasks in separate blocks. Each block of the baseline task contained 30 trials, whereas that of the operant task contained 90 trials comprising 30 repetitions of three possible action-outcome intervals. Observers completed two sets of randomly ordered task blocks (4 tasks × 2 = 8 blocks), resulting in 60 trials per condition (i.e., 480 trials in total). Each block began with three practice trials, which were not analyzed.

### Analysis

#### Behavioral data analysis

For each trial, an estimation error was calculated by subtracting the angle of the clock hand at the actual timing of the target event (keypress or tone) from the angle reported by the observer. These angle errors were transformed into temporal errors in ms. Positive (negative) numbers indicated overestimation (underestimation). For action and outcome timing, the mean errors were analyzed using one-way within-participant ANOVA with four conditions (baseline, 0 ms, 250 ms, and 500 ms operants). Moreover, I quantified the action and outcome binding by subtracting the average errors in the corresponding (key or tone) baseline errors from those in the operant conditions for each action-outcome interval length. Finally, intentional binding was calculated by summing up the amount of overestimation in action binding and underestimation in outcome binding. The binding effects were also analyzed using one-way ANOVA with three interval conditions. The *p* values below 0.05 were regarded as significant (two-tailed). Multiple comparisons were performed for significant main effects using Holm’s method.

#### Parameter recovery

For all the models, I simulated estimates for the same number of observers and trials (i.e., 480 trials for 76 observers) as in my experiment. To-be-recovered parameters were sampled from the uniform distributions whose ranges [lower, upper] were set to [0, 1] for $P(\xi =1)$, [0, 500] for ${\mu }_{AO}$, [0, 1000] for $\phi$, and [0, 1] for ${P}_{fr}$. I fitted each model to a simulated dataset and assessed Pearson’s correlations between the sampled and recovered parameters.

#### Model recovery

In the model recovery process, I systematically fitted each of the 12 computational models to the datasets produced during the parameter recovery process, yielding a comprehensive 12 by 12 matrix of model fits. For each simulated observer within the dataset, the model yielding the lowest AIC score was identified as the best fitting model. The frequency with which each model emerged as the best fit was then calculated and expressed as a proportion of the total simulated observers within each set of simulated data.

#### Model fitting and comparison

All the models shared fixed parameters regarding baseline estimates, which were drawn from observation. The BCI models shared $P(\xi =1)$ as a free parameter. Only the models with the coupling prior had an additional free parameter, ${\mu }_{AO}$. The threshold and false-report models each had one free parameter, $\phi$ and ${P}_{fr}$, respectively. Finally, the baseline model was a null model without a free parameter. I fitted each model to the data from each observer at the individual level and to the pooled data at the group level. The fitting was performed using MLE, defining the best parameter values that maximized the likelihood of a given model providing the observed estimates. The likelihood was obtained by simulating the model by generating 100,000 pairs of ${\tau }_{A}$ and ${\tau }_{O}$ (i.e., Monte Carlo simulation). The Monte Carlo simulations give predictions about how responses would be distributed under a certain model for a given condition. Using this distribution, one can calculate how well the actual response in a given trial can be predicted (i.e., likelihood) by each model. I searched for the best parameters with R^73^ and the R-package *pso*^74^. The possible parameter ranges were the same as those for parameter recovery. I then compared the different models’ abilities by reference to the best parameters based on the AIC, considering the difference in the parameter number. As a planned follow-up, I re-conducted the group-level fitting to the data excluding individuals whose responses were best accounted for by the false-report or baseline model.

### Supplementary Information

Supplementary Tables.

## Data Availability

The datasets and R sources for analyses during the current study are available in the Open Science Framework repository at https://osf.io/zsh8g/?view_only=dc09ba21700441498af9e909a2cf900e. The pre-registration of the experiment can be found in the AsPredicted repository (#97173) at https://aspredicted.org/KMQ_MHC.
